# GeneDB and Wikidata

**DOI:** 10.12688/wellcomeopenres.15355.2

**Published:** 2019-10-14

**Authors:** Magnus Manske, Ulrike Böhme, Christoph Püthe, Matt Berriman

**Affiliations:** 1 Parasites and Microbes, Wellcome Trust Sanger Institute, Cambridge, CB10 1SA, UK

**Keywords:** GeneDB, Wikidata, MediaWiki, Wikibase, genome, reference, annotation, curation

## Abstract

Publishing authoritative genomic annotation data, keeping it up to date, linking it to related information, and allowing community annotation is difficult and hard to support with limited resources.

Here, we show how importing GeneDB annotation data into Wikidata allows for leveraging existing resources, integrating volunteer and scientific communities, and enriching the original information.

## Introduction

The GeneDB website has presented genome annotation data from eukaryotic and prokaryotic pathogens ^1^ sequenced by the Wellcome Sanger Institute for more than 15 years. The underlying data are stored in a database, designed using the Chado ^2^ schema. The project was established to display genomes sequenced and annotated by the former Pathogen Sequencing Unit at the Sanger Institute, but over time the usage has changed. Now, genomes are stored and displayed if they are undergoing some level of curation or ongoing improvement. The site provides a way for curators and researchers to see changes to annotation long before those changes are integrated with other data types in a number of collaborating databases. To reflect the change of usage, where the website is often not the primary access point for many users, GeneDB has recently undergone a redesign and simplification ^3^. In particular, the web-based genome annotation tool Apollo ^4^ has been adopted as a major entry point for viewing genome data. While this delivers a structured, multi-track view of the genome and annotated genomic features (genes, ncRNAs, etc), the current version of Apollo has a limited capability for displaying the rich functional descriptions of individual genes that were a major feature of the previous GeneDB website.

Wikidata is a collaboratively edited, machine-readable and -writable knowledge base hosted by the Wikimedia Foundation, which also runs the collaboratively edited encyclopedia Wikipedia. Wikipedia has become the most accessed online encyclopedia and is unique in both its open, community-based editing, and a first port-of-call for public access to curated knowledge. Several bioinformatics projects make use of Wikipedia. The most successful of these is the Rfam project, where Wikipedia has been used to successfully manage free-text descriptions of RNA families ^5^ for over a decade. The Rfam-associated journal requires authors of new RNA families to create the matching Wikipedia page, tightly integrating Wikipedia into an entire field of research.

Wikidata currently contains 55 million items, which represent a superset of all Wikipedia article topics in over 300 languages, including biographical items, locations, species, artworks, scientific publications, etc. Amongst these items, Wikidata already stores human and mouse genes and proteins, as part of the Gene Wiki project ^6^, which originally started on Wikipedia ^7^, and many prokaryotic genes, as part of the WikiGenome project ^8^.

Wikidata offers various application programming interfaces (APIs) to read or write information in an automated way, including a query service using SPARQL, a query language for data on the Semantic Web ^9^. All these services are freely accessible by third-party users.

In the present study, we describe how we have exported the contents of GeneDB into Wikidata to ensure the long term sustainability of high value curated information and to make the annotated gene and protein information available to a wider audience. Within Wikidata, potentially anyone can contribute to the annotation, for instance by adding further external cross-references to third-party databases, linking gene and proteins to the scientific literature, or even short free-text descriptions. These community changes can be detected, checked, and, in appropriate cases, imported back into GeneDB.

We also describe utilising the Wikidata APIs to create a new version of the GeneDB website with content created solely based on Wikidata items. The design of the new GeneDB website closely mirrors the old one but now provides continuity and stability for incoming links from other websites. Furthermore, by building the site from Wikidata components, the new GeneDB website benefits from additional information and queries harvested from Wikidata.

## Methods

### Importing GeneDB into Wikidata

GeneDB exports its Chado database monthly into publicly accessible files ( ftp://ftp.sanger.ac.uk/pub/genedb/releases/) in general feature format version 3 (GFF3) and gene association file (GAF) format.

These files are regularly parsed by bespoke code to create or update Wikidata items, for both genes and their protein products.

This includes the addition of GO terms, as well as the creation and usage of Wikidata items about the scientific publications containing the respective findings.

An item about a gene (example: https://www.wikidata.org/wiki/Q19044775) or a protein on Wikidata consists of labels and aliases, descriptions, and a list of statements. Each statement is comprised of the following: a property from a community-controlled vocabulary (e.g., “chromosome”, “found in species”, “GeneDB ID”); a value, usually a plain string or a link to another Wikidata item, but also a date, a location, or a number, depending on the property; an optional list of qualifiers; and an optional list of references.

When updating an item, elements are added, altered, or removed on Wikidata if the current GeneDB information is different, and GeneDB is the authoritative source. All other elements of the Wikidata item remain unchanged during updates. Updates are performed automatically, utilizing the publicly exported GFF and GAF files based on Chado.

### Importing community changes from Wikidata into GeneDB

Community contributions on Wikidata can be divided into two parts. One part is the mass edit of items, by either bots (software-based robots that perform automated editing) or mass-editing tools. The other part are individual, usually manual edits, of low volume.

Only some edits are directly relevant to GeneDB; a new description of a protein in Dutch will not be imported into the Chado database, and can therefore be ignored. Likewise, the addition of external identifiers to sources not tracked by GeneDB can be ignored.

Individual edits that are both relevant to GeneDB, and not done by a Wikidata user on a “whitelist” of known, trusted users are summarized daily by an automated script, and sent to the GeneDB ticketing system for manual inspection. These changes are either ignored, reverted on Wikidata (e.g., vandalism), or imported into the GeneDB Chado database. The volume of such edits is quite low (~1/week) at this time, though we expect this to pick up with more members of the scientific community becoming aware of this venue into Wikidata.

### Implementation

The code to import and update Wikidata items was written in Rust ^10^ (rustc 1.36.0), using (amongst others) the rust-bio crate ^11^ for GFF and GAF reading. Rust was chosen as a language for its speed, security, low resource consumption, and available crates (libraries) to build on. Some of these crates, such as MediaWiki and Wikibase (Wikidata) API handling, were (co-)developed by the corresponding author independently. See software availability for source code ^12, 13^

### Operation

The Wikidata import code will run on any platform that Rust can be compiled for. Additional requirements are an internet connection, and login information for a Wikidata bot account.

The website operates client-side using JavaScript, and can be deployed on any standard web server. Besides Wikidata, no additional server-side support is required.

## Results/Use cases

At this time (26/07/2019), 409,219 Wikidata items for genes (including 9,031 pseudogenes), and an additional 397,979 items for proteins, have been created ( http://tinyurl.com/y6wpfyrn), covering 45 taxa.

**Figure 1.**
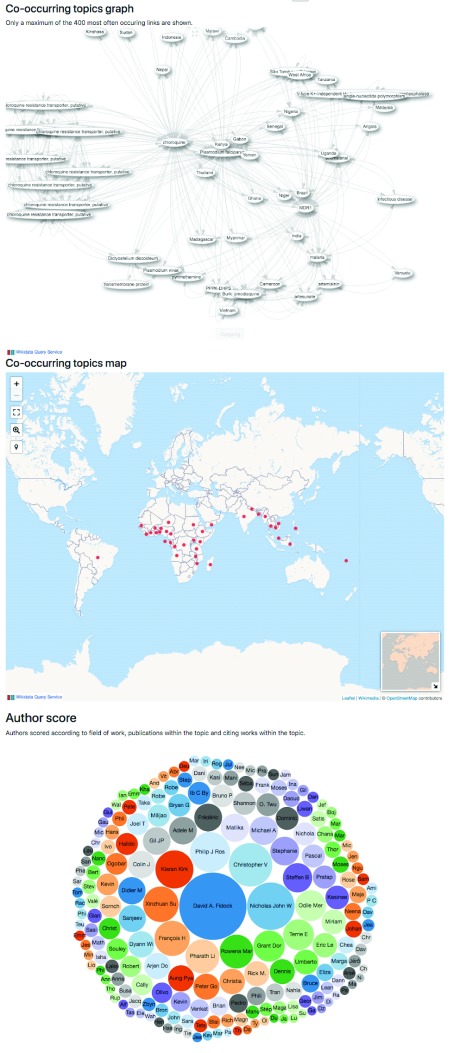
Part of the Scholia tool website about the PfCRT gene ( https://tools.wmflabs.org/scholia/topic/Q19044775). These figures show connected topics, connected locations on a map, and the most prolific authors for this topic, respectively. All figures are generated live from Wikidata via its various APIs. All this information, and more, results from linking scientific publications to the genes in Wikidata.

By exporting from GeneDB (Chado) into Wikidata, the data are intricately integrated into its ecosystem creating new functions with minimal project-specific development; for instance, links to and between publications, which in turn link to authors, institutions, etc. These connections between items allow for complex queries that were not possible before ( Figure 1).

### Gene pages

To replicate the way genes were represented on the previous version of the GeneDB website, a pure HTML/JavaScript site using vue.js was created. JavaScript components are written as modules. All pages and components are designed to work on both desktop and mobile. Web pages for genes, proteins, species, chromosomes, GO term ^14^ queries, and searches are generated on-the-fly utilising the Wikidata API and SPARQL interface, and Wikidata serves as the only back-end for these pages.

**Figure 2.**
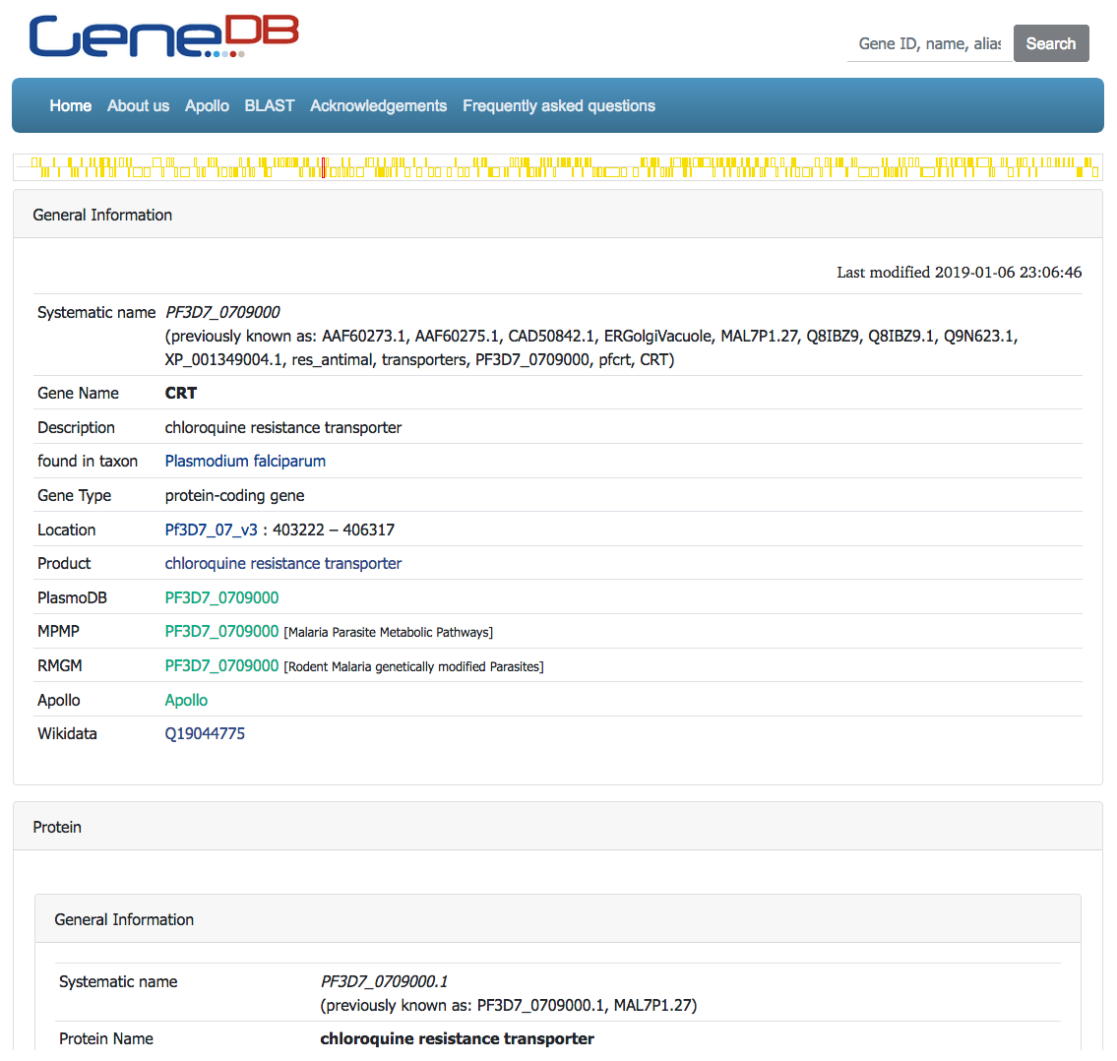
Gene information on GeneDB ( https://www.genedb.org/gene/PF3D7_0709000). All information comes exclusively from Wikidata.

Each gene item, and its associated protein item(s), can be viewed on a separate page ( Figure 2), that is rendered on-the-fly. This rendering includes a map of the gene on the chromosome, names, IDs, descriptions, links to other web resources (both from Wikidata statements, and auto-generated based on species and gene ID), a link to the Apollo browser view of the gene, a list of known orthologs.

**Figure 3.**
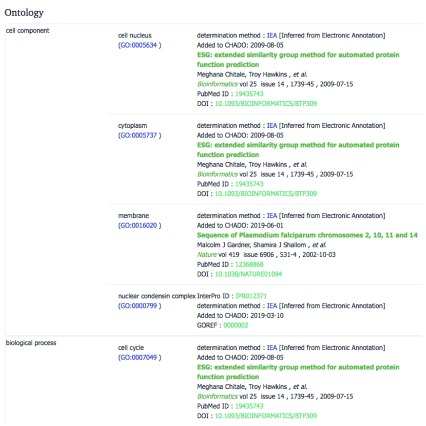
GO term ontology ( https://www.genedb.org/gene/PF3D7_1135600), with sources.

Below that, each protein encoded by the gene is listed, as well as the known GO term ontology, complete with evidence and publication links, where available ( Figure 3).

If Wikidata contains items about publications that have the gene or protein as a “main subject”, these publications are listed at the bottom of the page. This is an example of additional, on-topic information that Wikidata provides on top of the GeneDB dataset.

Gene/protein pages link out to other web-based databases via Wikidata-stored (e.g., UniProt) or computed (e.g., PlasmoDB) URLs. GO terms show supporting information, including citations of, and links to, the original publications. Also, a list of all publications on Wikidata with the respective gene as a subject is available on both GeneDB (example: https://www.genedb.org/gene/PF3D7_1200600) and the Scholia tool (example: https://tools.wmflabs.org/scholia/topic/Q18971176).

### Other functionality

The GeneDB search function utilizes Wikidata search, letting users find genes by name, alias, ID, and related information, across all covered species. The search will only return genes on Wikidata with a GeneDB identifier.

**Figure 4.**
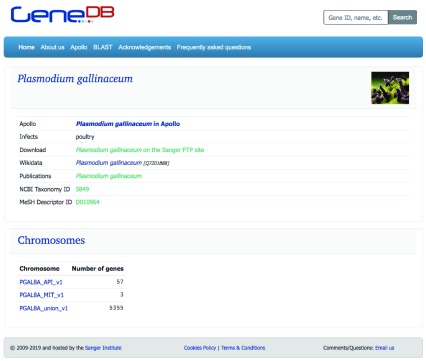
Species page for *Plasmodium gallinaceum*, using information and image from Wikidata.

Each species in GeneDB has its own page ( Figure 4), showing basic information about the species, links to other web resources, to the Apollo browser, and a list of chromosomes linking to the genes located there.

**Figure 5.**
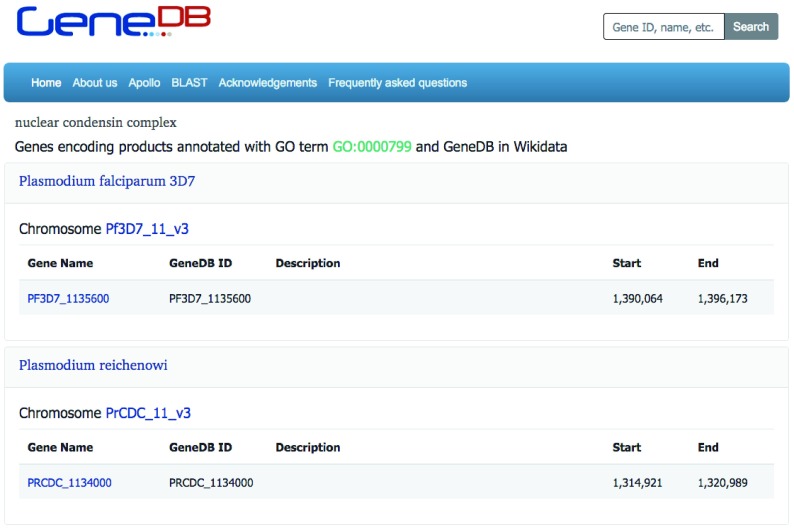
Genes for proteins with a specific GO term ( https://www.genedb.org/#/go/GO:0000799), in all species. Results from Wikidata, limited to genes with a GeneDB ID.

Genes/proteins annotated with specific GO terms can be listed, grouped by species ( Figure 5). These lists are linked from every GO term on a gene/protein page.

## Discussion

Wikidata has provided GeneDB with a venue to host and publish its data, and to invite community edits, without giving up on GeneDB’s curation authority. The simplified maintenance of the HTML/JS-only GeneDB website, compared to a previous one that combined a frontend and backend solution, frees technical and personnel resources. Linking genes and proteins to, and from, other Wikidata items allows for novel methods of querying the data, and for new questions to be asked. Publishing on Wikidata also exposes the data in new ways and potentially engages with a broader public audience.

For sustained operation, we are working on a unified, curated update mechanism that takes user-generated input from Wikidata, Apollo, and Artemis ^15^, and lets professional curators validate the changes before feeding them back to the Chado database. Changes on Wikidata that are not curated by the GeneDB project may be displayed on the GeneDB website without curation.

## Data availability

### Source data

**Chado-based GFF and GAF files**

ftp://ftp.sanger.ac.uk/pub/genedb/releases/

**Accession numbers in use cases**

GeneDB IDs

Pathogen genomic data from GeneDB, Accession number PF3D7_0709000. https://identifiers.org/genedb:PF3D7_0709000

Pathogen genomic data from GeneDB, Accession number: PF3D7_1200600. https://identifiers.org/genedb:PF3D7_1200600

Pathogen genomic data from GeneDB, Accession number: PF3D7_1135600. https://identifiers.org/genedb:PF3D7_1135600

Wikidata items

Genomic data for VAR2CSA from Wikidata, Accession number: Q18971176. https://identifiers.org/wikidata:Q18971176

Genomic data for CRT from Wikidata, Accession number: Q19044775. https://identifiers.org/wikidata:Q19044775

*Plasmodium gallinaceum* data from Wikidata, Accession number: Q7201888. https://identifiers.org/wikidata:Q7201888

GO terms

Gene Ontology entry for nuclear condensin complex, Accession number: GO:0000799. https://identifiers.org/GO:0000799

### Underlying data

All data underlying the results are available as part of the article and no additional source data are required.

All data is in the public domain (GeneDB) ^16^ or CC-0 (Wikidata), which are effectively equivalent.

## Software availability

GeneDBot, the code that updates Wikidata from Chado:

Source code: https://github.com/sanger-pathogens/genedbot_rs

Archived source code at time of publication: http://doi.org/10.5281/zenodo.3352001 ^12^

License: GPL v3.0

The HTML/JS code for the GeneDB website (Wikidata display code is mostly in the htdocs/wd directory):

Source code: https://github.com/sanger-pathogens/GeneDB-static

Archived source code at time of publication: http://doi.org/10.5281/zenodo.3352003 ^13^

License: GPL v3.0

## References

[1] Logan-Klumpler FJ De Silva N Boehme U: GeneDB--an annotation database for pathogens. *Nucleic Acids Res.* 2012;40(Database issue):D98–D108. 10.1093/nar/gkr1032 22116062 PMC3245030

[2] Mungall CJ Emmert DB, FlyBase Consortium: A Chado case study: an ontology-based modular schema for representing genome-associated biological information. *Bioinformatics.* 2007;23(13):i337–i346. 10.1093/bioinformatics/btm189 17646315

[3] Böhme U Otto TD Sanders M: Progression of the canonical reference malaria parasite genome from 2002–2019 [version 1; peer review: 2 approved, 1 approved with reservations]. *Wellcome Open Res.* 2019;4:58. 10.12688/wellcomeopenres.15194.1 31080894 PMC6484455

[4] Dunn NA Unni DR Diesh C: Apollo: Democratizing genome annotation. *PLoS Comput Biol.*Darling AE, editor.2019;15(2):e1006790. 10.1371/journal.pcbi.1006790 30726205 PMC6380598

[5] Daub J Gardner PP Tate J: The RNA WikiProject: community annotation of RNA families. *RNA.* 2008;14(12):2462–2464. 10.1261/rna.1200508 18945806 PMC2590952

[6] Waagmeester A Schriml L Su A: Wikidata as a linked-data hub for Biodiversity data. Pensoft Publishers; *Biodiversity Information Science and Standards.* 2019;3:e35206 10.3897/biss.3.35206

[7] Burgstaller-Muehlbacher S Waagmeester A Mitraka E: Wikidata as a semantic framework for the Gene Wiki initiative. *Database (Oxford).* 2016;2016: pii: baw015. 10.1101/032144 26989148 PMC4795929

[8] Putman TE Lelong S Burgstaller-Muehlbacher S: WikiGenomes: an open web application for community consumption and curation of gene annotation data in Wikidata. *Database (Oxford).* 2017;2017(1): 10.1093/database/bax025 28365742 PMC5467579

[9] Segaran T Evans C Taylor J: Programming the Semantic Web.O’Reilly Media;2009 Reference Source

[10] Contributors to Wikimedia projects: Rust (programming language) - Wikipedia. In: Wikimedia Foundation, Inc. 2010; [cited 15 May 2019]. Reference Source

[11] Köster J: Rust-Bio: a fast and safe bioinformatics library. *Bioinformatics.* 2016;32(3):444–446. 10.1093/bioinformatics/btv573 26446134

[12] Manske M: sanger-pathogens/genedbot v1.0.0 (Version v1.0.0). *Zenodo.* 2019 10.5281/zenodo.3352001

[13] pathdbpi Offord V Manske M: sanger-pathogens/GeneDB-static v1.0.0 (Version v1.0.0). *Zenodo.* 2019 10.5281/zenodo.3352003

[14] Ashburner M Ball CA Blake JA: Gene ontology: tool for the unification of biology. The Gene Ontology Consortium. *Nat Genet.* 2000;25(1):25–29. 10.1038/75556 10802651 PMC3037419

[15] Carver T Harris SR Berriman M: Artemis: an integrated platform for visualization and analysis of high-throughput sequence-based experimental data. *Bioinformatics.* 2012;28(4);464–9. 10.1093/bioinformatics/btr703 22199388 PMC3278759

[16] The data release policy on GeneDB, placing all data in the public domain. Reference Source

