# Supplementary material for: GeneDB and Wikidata
Source: Wellcome Open Res. 2019 Oct 14;4:114. Originally published 2019 Aug 1. [Version 2] doi: 10.12688/wellcomeopenres.15355.2 (PMC6823904; doi:10.12688/wellcomeopenres.15355.2)
Supplement: Peer review history

# Peer review: Reviewer response for version 2

**DOI:** 10.21956/wellcomeopenres.16983.r36761

**Published:** 2019-10-30

**Authors:** Andra Waagmeester

**Affiliations:** 1 Micelio, Antwerp, Belgium

The authors addressed the comments.

I confirm that I have read this submission and believe that I have an appropriate level of expertise to confirm that it is of an acceptable scientific standard.

# Peer review: Reviewer response for version 2

**DOI:** 10.21956/wellcomeopenres.16983.r36762

**Published:** 2019-10-15

**Authors:** Sebastian Burgstaller-Muehlbacher

**Affiliations:** 1 Max Perutz Labs, Vienna Biocenter, Medical University of Vienna, Vienna, Austria

The authors modified the original manuscript as suggested, no further comments from my side.

I confirm that I have read this submission and believe that I have an appropriate level of expertise to confirm that it is of an acceptable scientific standard.

# Peer review: Reviewer response for version 1

**DOI:** 10.21956/wellcomeopenres.16772.r36100

**Published:** 2019-08-27

**Authors:** Andra Waagmeester

**Affiliations:** 1 Micelio, Antwerp, Belgium

This paper describes work done in linking data from the GeneDB website in Wikidata. GeneDB captures genome annotation data. The article also describes how a feedback loop is created enriching GeneDB with content from Wikidata. This is an exciting development where the UI directly leverages content from Wikidata, instead of using in-house caching databases.

 I have some minor comments. 

1. Wikidata uses a CC0 license on captured data. It is not clear if this is compatible with the applicable Guidelines on the use of data in publications on GeneDB ( https://www.sanger.ac.uk/legal/). The terms for usage require explicitly attribution, which CC0 does not. I am curious how the authors solved this. A section on legalities of sharing GeneDB on Wikidata would be a welcome addition. 
2. "The most successful of these is the Rfam project, where Wikipedia has been used to successfully manage free-text descriptions of RNA families ^5^ for over a decade." Why is this the most successful project, who are the second and third runner up?

I confirm that I have read this submission and believe that I have an appropriate level of expertise to confirm that it is of an acceptable scientific standard, however I have significant reservations, as outlined above.

# Response

**Published:** 2019-10-10

**Authors:** Magnus Manske

Thank you for your review, and your constructive suggestions. In reply to your individual points:

- Data from GeneDB is in the public domain, which is compatible with CC-0. The paper and the "data release" page of the GeneDB website have been updated accordingly.
- Regarding Rfm, we use "most successful" to describe the uptake of Wikipedia as a central part of the RNA families journal, and thereby, scientific community. This has been added to the manuscript.

# Peer review: Reviewer response for version 1

**DOI:** 10.21956/wellcomeopenres.16772.r36099

**Published:** 2019-08-20

**Authors:** Sebastian Burgstaller-Muehlbacher

**Affiliations:** 1 Max Perutz Labs, Vienna Biocenter, Medical University of Vienna, Vienna, Austria

The authors describe a new implementation and technical foundation of GeneDB, a database for eukaryotic and prokaryotic parasites. As a new technical foundation, the authors use Wikidata, an open, semantic web enabled graph database operated by the Wikimedia Foundation. To integrate GeneDB data into Wikidata, the authors wrote bots (robots/database clients) using the language Rust and imported GeneDB genes, proteins and Gene Ontology annotations into Wikidata. Furthermore, they created a website which renders the data imported into Wikidata in a comprehensive and more user-friendly way than a raw Wikidata data item would. Also, they integrate genome track views using Apollo.

 Overall, the study opens up a new dataset to the public domain entirely. Data are imported and made available via the Wikidata API and the Wikidata SPARQL endpoint, allowing users of the data powerful queries. Having these data available and easily accessible to the parasite research community worldwide seems crucial. Easy access is also enabled by the web interface the authors implemented.

 Moreover, due to the nature of Wikidata, contributions by the (research) community are relatively easy to accomplish. The authors also provide a reasonable approach for feeding back (research) community contributions into GeneDB, while avoiding vandalism.

 Integration with the Apollo genome browser also provides a genome view, typical for a gene/genome centered database. However, the authors should describe in some more detail how they setup Apollo and where Apollo gets its genome tracks from (e.g. DNA sequences). This is relevant as such data typically cannot be stored in Wikidata and thus need to be drawn from a different source.

 The manuscript is well-written, however the introduction should also mention the work of Putman *et al.* (2017 ^1^), who follow a similar technical approach but for a different set of genomes. The discussion is rather brief and could profit from addressing the authors long-term maintenance plans, especially regarding curation of the inflow of community contributions to GeneDB.

 In summary, this work by Manske *et al.* is very well performed, except for the minor points mentioned above. It’s an important data release for the parasite research community and enables, for the first time, collaborative work on genomic data integration and data annotation within this community. This should lower greatly the hurdles for worldwide collaboration on alleviating the severe diseases associated with many of the parasites in GeneDB.

 Ad citation 7 of the manuscript: This preprint has now been published as Burgstaller-Muehlbacher *et al.* (2016 ^2^).

I confirm that I have read this submission and believe that I have an appropriate level of expertise to confirm that it is of an acceptable scientific standard.

## References

[1] : WikiGenomes: an open web application for community consumption and curation of gene annotation data in Wikidata. *Database*.2017;2017: 10.1093/database/bax025 10.1093/database/bax025 PMC546757928365742

[2] : Wikidata as a semantic framework for the Gene Wiki initiative. *Database (Oxford)*.2016;2016: 10.1093/database/baw015 10.1093/database/baw015 26989148PMC4795929

# Response

**Published:** 2019-10-10

**Authors:** Magnus Manske

Thank you for your review, and your constructive suggestions. In reply to your individual points:

- The Apollo part of the GeneDB website is not part of this publication. Please see Böhme, Otto et al. (2019) for details.
- The reference to Putman et al. (2017) has been added.
- Some discussion of long-term sustainability has been added.
- The Burgstaller-Muehlbacher citation has been updated.
